# Supplementary material for: Dietary Advanced Glycation Endproducts Induce an Inflammatory Response in Human Macrophages in Vitro
Source: Nutrients. 2018 Dec 2;10(12):1868. doi: 10.3390/nu10121868 (PMC6315629; doi:10.3390/nu10121868)
Supplement: Supplementary file 1 [file nutrients-10-01868-s001.pdf]

Table S1. Target compounds and selected instrumental parameters for quantification of each compound by UPLC-(ESI+)MS/MS.

**Compound**	**Precursor > product ion (*m/z*)**	**Cone voltage (V)**	**Collision energy (eV)**	**Internal Standard used**
CML	205.2 > 84.2	20	20	CML-d2
	205.2 > 130.2	20	12	
CEL	219.2 > 84.2	20	20	CEL-d4
	219.2 > 130.2	20	10	
MG-H1	229.2 > 114.1	20	13	MG-H1-d3
	229.2 > 166.2	20	13	
Pentosidine	379.4 > 135.1	20	40	MG-H1-d3
	379.4 > 187.1	20	35	

Note: the underlined precursor > product ion transition was used for quantification, the *italic* precursor > product ion transition was used for confirmation.

Table S2. Instrumental detection and quantitation limits (pg on column) and method detection and quantitation limits (pg on column)

**Compound**	**IDL (pg on column)**	**IQL (pg on column)**	**MDL (µg/ml)**	**MQL (µg/ml)**
CML	0.5	1.5	0.001	0.003
CEL	1.0	3.5	0.01	0.03
MG-H1	1.7	5.7	0.03	0.1
Pentosidine	6.4	21	1.2	4
